# On-line parameter identification of the lumped arterial system model: A simulation study

**DOI:** 10.1371/journal.pone.0236012

**Published:** 2020-07-10

**Authors:** Feng Huang, Shunv Ying

**Affiliations:** 1 College of Metrology & Measurement Engineering, China Jiliang University, Hangzhou, China; 2 The Affiliated Stomatology Hospital, Zhejiang University School of Medicine, Hangzhou, China; King's College London, UNITED KINGDOM

## Abstract

A lumped model of the arterial system has been used in constructing a hybrid mock loop due to its real-time response. However, the parameters of the model are always from a general case and not adapted to a specific patient. In this study, we focused on on-line parameter identification of the lumped model of the arterial system that could be used for a specific patient. A five-element lumped arterial model is adopted in this study, in which the five parameters are to be determined. The aortic flow rate and the venous pressure are chosen as the inputs of the model, and aortic pressure as the output. An iterative optimization based on the established state space equations of the five-element model is used to seek the best parameter values by minimizing the difference between the model prediction and the previously obtained aortic pressure. The method is validated using simulated data from a complete numerical cardiovascular model. Results show that the method can track the dynamic variation of the parameters very well. Finally, a sensitivity analysis of the model parameters is conducted to interpret the effect of parameter changes. The good performance of the identification demonstrates the potential application of this method to customize a hybrid mock loop for a specific patient or clinically monitor the arterial vessel characteristics in real time.

## Introduction

The lumped model of the arterial system is a simplification of realistic arterial system. It usually appears as an important part of a cardiovascular model. Due to its good characteristics and simplicity, it has been used in real-time hybrid mock loops (HMLs) successfully [1,2]. A hybrid mock loop (HML) is constructed based on the “hardware in the loop” conception that combines a numerical cardiovascular model and physical hardware together. Compared with traditional hydraulic mock loops [3–5], the HML has advantages of easy modification and parameter adjustment in the numerical model. It means the HML could reproduce multiple physiologic conditions by modifications only in software. In this sense, it would be convenient to create a HML for a specific patient and then modify the model parameters for the next patient.

In the HML, the hardware mainly provides an interface between the test cardiovascular devices and the numerical cardiovascular model. Specifically, the blood pressures at relevant sites are simulated by the numerical circulation model and then physically produced by the hardware. These pressures provide an “in-circulation-similar” working condition for the test cardiovascular devices, and afterwards, the devices will generate “the same” flow as if they were implanted in a body. This flow data is sent back into the numerical circulation model to obtain new calculated blood pressures. The test cardiovascular devices interact with the numerical circulation model via this feedback loop.

As the main load of the heart, the model of the arterial system is one of the most important part of the numerical cardiovascular model, especially for a HML aiming to evaluate left ventricular assist devices or artificial aortic valves. Compared with the more complicated distributed models, the lumped model of the arterial system is easier to use and requires less computational resources, which is quite favorable for real-time running of a HML.

The lumped parameter model of the arterial system was proposed by Frank [6], which is known as the two-element Windkessel model with only resistance and compliance parameters. After that, researchers improved the model by adding more elements. The five-element model tries to approximate reality better by splitting the lumped compliance of the entire arterial system into two components separated by an inertance. Although the five elements do not correspond to specific vessels, they as a whole demonstrate a good arterial impedance characteristic reproduction over a wide frequency range that covers the physiological range of heart rate [7,8] and has a good balance between performance and complexity. However, the parameters of the five-element model are always the same in previous studies [1,2,8]. It would be interesting to customize the model for a specific person. In that sense, as for a HML, it means testing of a cardiovascular device for a specific patient who will receive it. For this purpose, it is desirable to identify the parameters of the model according to the hemodynamics of the specific patient.

Although some studies [9,10] used large-scale parameters estimation of the cardiovascular system in which the number of the parameters is large, this resulted in a high computational cost; there are fewer studies focusing on the identification of the simple lumped arterial system model. As for the application in a HML, a simple lumped arterial system model is usually enough because a more elaborate output of the model is harder to realize physically in practice. Additionally, since the five-element model is simple, the parameter identification will be accurate and fast enough (within seconds), while a more complicated arterial system model identification usually takes more than 24 h [10]. In another word, the parameter identification of the lumped arterial model could be carried out on-line to trace parameter variation during short-period physiological state changes. Therefore, to provide a thorough investigation of the identification of the simple five-element arterial system model is also necessary.

**Fig 1:**
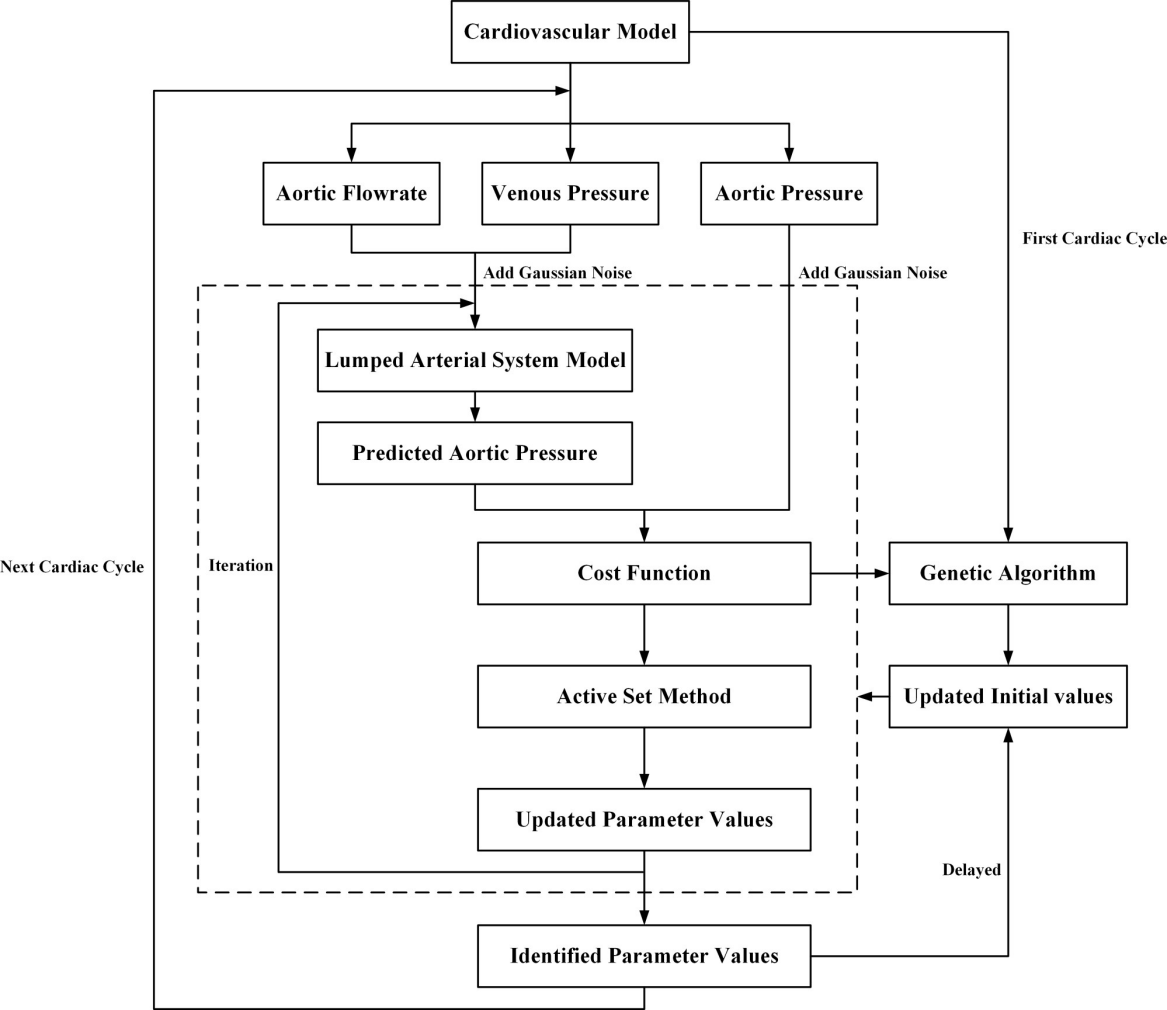
The framework of the on-line parameter identification.

In this study, an on-line method to identify the parameters of the five-element arterial model based on iterative optimization method is presented. The state space equations of the model were built to be used in the optimization process, in which the aortic flow rate and the venous pressure were the inputs and the aortic pressure was the single output. The optimization minimized the difference between the model prediction output and the actual data. The on-line parameter identification method was validated in a complete numerical cardiovascular model, and good results were obtained. To avoid manual selection of the initial values of the parameters to be identified, we conducted an extra genetic algorithm at beginning to get a rough parameter identification result to be used as the initial values. The framework of this research is depicted in Fig 1, with all the details described in the Methods section. A sensitivity analysis of the model parameters was also carried out to show the effect of each parameter change.

## Methods

### Cardiovascular model

**Fig 2:**
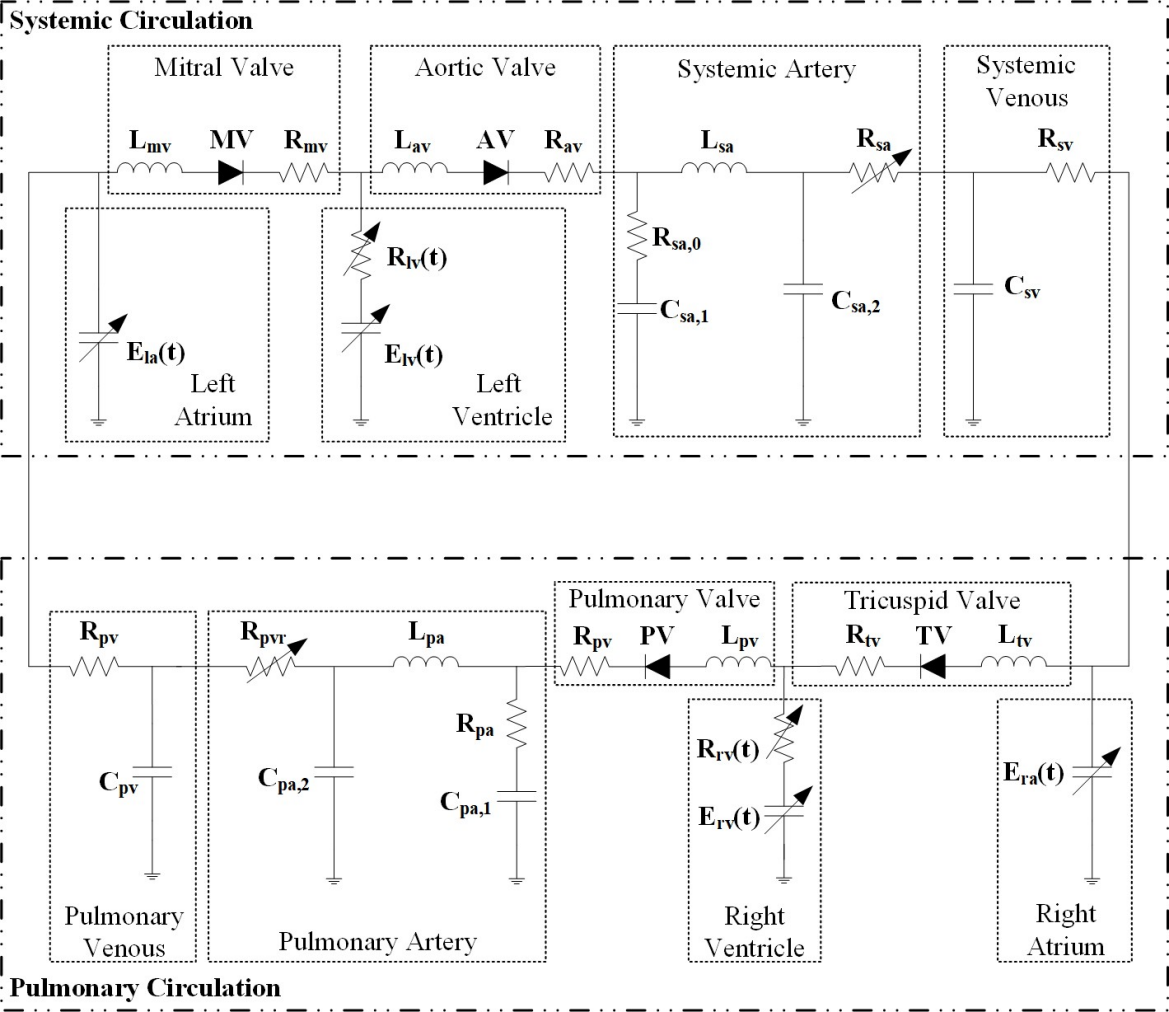
The complete cardiovascular system model used for validating the identification algorithm.

A complete numerical model of the cardiovascular system from a previous study [11], shown in Fig 2, is adopted in this study to provide a platform for validating the on-line identification algorithm. The cardiovascular model includes both systemic and pulmonary circulation. The ventricular and atrial chambers are described using the nonlinear time-varying elastance model, with different elastance values for ventricles and atria. All the heart valves, with the main function of ensuring the one-way blood flow, are modeled by ideal diodes coupled with resistance and inertance. The arterial system, either systemic or pulmonary, applies the five-element lumped parameter model, but with different parameter values. The systemic and pulmonary venous systems are both characterized simply by lumped resistance and lumped compliance. More details and parameter values could be referred to the reference [11,12].

### Lumped arterial system model

**Fig 3:**
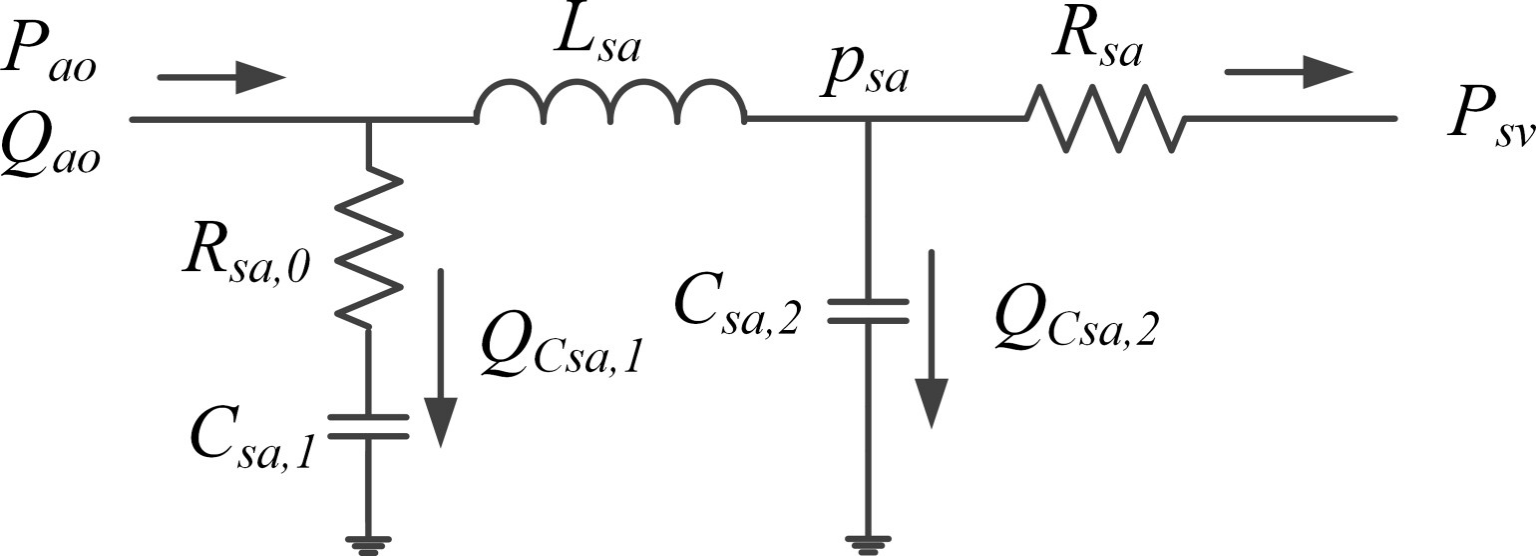
The five-element lumped model of the arterial system. ***p*_*ao*_**, aortic pressure; ***Q***_***ao***_, aortic flowrate; ***p***_***sv***_, systemic venous pressure; ***R***_***sa*,0**_, characteristic systemic resistance; ***R***_***sa***_, systemic arterial resistance; ***L***_***sa***_, systemic arterial inertance; ***C***_***sa*,1**_ and ***C***_***sa*,2**_, two components of the arterial compliance; $Q_{C_{sa,1}}$ and $Q_{C_{sa,2}}$, flowrates through the two compliances; ***p***_***sa***_, pressure before pressure drop of the systemic arterial resistance.

The lumped systemic arterial system model used in the cardiovascular model is a five-element Windkessel model, which is detailly depicted in Fig 3. It includes two resistances, two compliances, and one inertance. The inertance splits the lumped compliance of the entire arterial system into two components to approximate reality better, compared with the classic Windkessel model that only has one resistance and one compliance [7]. The inputs of this model are the flow rate *Q*_*ao*_ at the aortic root and the systemic venous pressure *p*_*sv*_, while the output is the aortic pressure *p*_*ao*_.

The differential equations describing this lumped arterial system model are as follows.

$$C_{sa,1}∙\frac{d(p_{ao}-R_{sa,0}∙Q_{C_{sa,1}})}{dt}=Q_{C_{sa,1}}$$ (1)

$$L_{sa}∙\frac{d(Q_{ao}-Q_{C_{sa,1}})}{dt}=p_{ao}-p_{sa}$$ (2)

$$C_{sa,2}∙\frac{dp_{sa}}{dt}=Q_{C_{sa,2}}$$ (3)

All the notations are shown in Fig 3. There are three energy storage elements in the model, and three differential equations are used to describe it. Finally, the pressure drop across the systemic arterial resistance *R*_*sa*_ is used to close the description of the model.

$$R_{sa}∙(Q_{ao}-Q_{C_{sa,1}}-Q_{C_{sa,2}})=p_{sa}-p_{sv}$$ (4)

Substituting Eq (4) into Eq (3) and eliminating $Q_{C_{sa,2}}$, then rearranging the above equations, the state space description of the model is obtained:

$$\begin{array}{l} \dot{X}=AX+BU\\ Y=CX \end{array}$$ (5)

where *X*∈*R*^3×1^ and *Y*∈*R*^1×1^ are the state vector and the output vector, respectively, and *U*∈*R*^3×1^ is the input vector. They are represented as follows, where $\dot{Q_{ao}}$ is the derivative of the aortic flow rate and *T* is the transpose of the matrix. Other notations could be found in the legend of Fig 3.

$$X={\left[p_{ao}\;p_{sa}\;Q_{C_{sa,1}}\right]}^{T},\;Y=p_{ao},\;U={\left[Q_{ao}\;\dot{Q_{ao}}\;p_{sv}\right]}^{T}$$ (6)

Here, *A*∈*R*^3×3^, *B*∈*R*^3×3^, and *C*∈*R*^1×3^ are the state matrix, the input matrix, and the output matrix, respectively, which are expressed in the following forms.

$$\begin{array}{l} A=\left[\begin{array}{ccc} -\frac{R_{sa,0}}{L_{sa}} & \frac{R_{sa,0}}{L_{sa}} & \frac{1}{C_{sa,1}}\\ 0 & -\frac{1}{R_{sa}∙C_{sa,2}} & -\frac{1}{C_{sa,2}}\\ -\frac{1}{L_{sa}} & \frac{1}{L_{sa}} & 0 \end{array}\right]\\ B=\left[\begin{array}{ccc} 0 & R_{sa,0} & 0\\ \frac{1}{C_{sa,2}} & 0 & \frac{1}{R_{sa}∙C_{sa,2}}\\ 0 & 1 & 0 \end{array}\right]\\ C=[\begin{array}{ccc} 1 & 0 & 0 \end{array}] \end{array}$$ (7)

### On-line parameter identification

There are five unknown parameters in the above lumped systemic arterial system model that need to be identified, corresponding to the five elements. The vector form of the identified parameters Θ∈*R*^5×1^ is defined as

$$\Theta ={\left[R_{sa,0}\;R_{sa}\;C_{sa,1}\;C_{sa,2}\;L_{sa}\right]}^{T}$$ (8)

To determine Θ, we applied an iterative optimization method. The input vector *U* obtained from the cardiovascular model is buffered for every cardiac cycle and then fed into the state space model with initial parameter values. The iterative optimization process will minimize the squared error between the predicted outputs of the state space model and the actual output from the cardiovascular model by altering the parameter vector Θ. The identified Θ is then used as the initial parameter values for the next cardiac cycle to obtain an updated identified value of Θ. In this way, the lumped parameters of systemic arterial system will be identified on-line.

$$\min_{\Theta }\;J=\int {\left(Y(t)-\tilde{Y}(t,\Theta )\right)}^{2}dt$$ (9)

Here, J is the cost function and *Y*(*t*) is the actual output from the cardiovascular model, while $\tilde{Y}(t,\Theta )$ is the output of the state space model. To use the discrete data from simulation, we discretized Eqs (5) and (6). Considering the physiological meaning of the parameters in Θ, their values have maximum and minimum physiological values, which are given as Θ_*lb*_ and Θ_*ub*_. Combining these, the whole parameter identification could be represented as

$$\begin{array}{l} \min_{\Theta }\;J=\sum_{k=1}^{m}h{\left(Y_{k}-{\tilde{Y}}_{k,\Theta }\right)}^{T}(Y_{k}-{\tilde{Y}}_{k,\Theta })\\ s.t.\;{\tilde{X}}_{k+1,\Theta }={\tilde{X}}_{k,\Theta }+hA{\tilde{X}}_{k,\Theta }+hBU_{k}\\ \;{\tilde{Y}}_{k,\Theta }=C{\tilde{X}}_{k,\Theta },\;k=1,2,3\cdots m\\ \;\Theta_{lb}\leq \Theta \leq \Theta_{ub} \end{array}$$ (10)

where *m* is the number of data during every cardiac cycle, *k* is the discretization point, and *h* is the time interval between two consecutive data points.

Synthesizing the data from the literature [7,8,12,13], physiologically reasonable maximum and minimum physiological values of the parameters are chosen according to Table 1, which are intentionally chosen somewhat outside of the normal physiological range. Accordingly, these limits, which are also used to constrain the optimal searching domain, i.e., Θ_*lb*_ and Θ_*ub*_ in Eq (10), are given by

$$\begin{array}{l} \Theta_{lb}={\left[0.01\;0.5\;0.1\;0.1\;0.00001\right]}^{T}\\ \Theta_{ub}={\left[0.5\;2.0\;2.0\;2.0\;0.1\right]}^{T} \end{array}$$ (11)

**Table 1:** Maximum and minimum physiological values of the parameters.

Parameter	Value			Unit
	Maximum values	Normal^#^	Minimum values	
*R*_*sa*,0_	0.5	0.1	0.01	*mmHg*∙*s*/*mL*
*R*_*sa*_	2.0	1.0	0.5	*mmHg*∙*s*/*mL*
*C*_*sa*,1_	2.0	0.9	0.1	*mL*/*mmHg*
*C*_*sa*,2_	2.0	0.25	0.1	*mL*/*mmHg*
*L*_*sa*_	0.1	0.0003	0.00001	*mmHg*∙*s*^2^/*mL*

^#^The normal values are from the literature by Colacino [12].

A numerical method of the nonlinear minimum optimization was adopted to solve the above problem in MATLAB/Simulink (Mathworks Inc., MA, United States). The optimization applied the active set method which run in a fast speed for a small-scale problem and thus was suitable for the on-line identification [14]. The active set includes all the equality constraints and those that are on the inequality constraint boundaries. The active set was active because the constraints could be added or removed during iterations. By means of this the optimization was converted to the equality problem defined by the active set in an iteration, which could be solved with the normal Lagrange multiplier method. The iterations stopped when the found solution satisfied the KKT condition. The active set method reduces the complexity of the optimal search and has a fast speed. More details of the active set method could be found in the reference [14]. The termination tolerance of the cost function and the maximum number of iterations are set as 10^−4^ and 100, respectively.

### Initial values

The iterative optimization needs setting the initial values of the parameters to be identified. The initial values are of great importance to most of the iterative optimization problems because the minimization searching algorithm usually converges to local minima instead of global minima. During every cardiac cycle, the initial values of the parameters to be identified were from the identification result in the previous cardiac cycle, in which way the used initial values were always near the local minima and thus the iterative optimization would be fast converged. In order to give the initial values at the beginning, in other words at the first cardiac cycle of the identification, a genetic algorithm was introduced to find the global minima, and the obtained parameter values were then set as the initial values for the subsequent cardiac cycle.

The genetic algorithm is a stochastic optimization algorithm which was inspired by natural selection and natural genetics [15]. The definition domain of each parameter to be identified was divided into appropriate intervals and then coded as a binary string, after which combined together in sequence of parameter. In this way a specific value of the parameter vector was mapped to a string, which is called an individual in a population. The optimization started with a population of 100 individuals, where every bit of the individual string was initialized randomly. After that, genetic operators such as reproduction, crossover and mutation were performed iteratively to create a new generation of population. During each iteration, the individuals in the population were selected according to their cost function value described in Eq (10). The algorithm stops when either the average relative change of the cost function value was small or the maximum number of iterations was reached.

The genetic algorithm is a slow but global optimization algorithm compared to the active set nonlinear optimization method. It guarantees the necessary feasible starting point of the active set method. By combining these two optimization methods together, the speed and the global convergence are ensured in the on-line identification.

### Parameter sensitivity

To see the sensitivity of the lumped arterial system to the parameter variations, we carried out a sensitivity analysis. The sensitivity function of the system to every parameter is defined as

$$S_{\Theta_{i}}^{G}(s)=\frac{\partial G(s,\Theta )}{\partial \Theta_{i}}{|}_{\Theta_{0}},i=1,2,3,4,5$$ (12)

where *i* represents every parameter, *s* is the Laplace variable, and Θ_0_ is the specific point near which the sensitivity analysis is based. *G* is the transfer function of the arterial system from multiple inputs to the single output (MISO, as defined in Eq (6)), which is converted from the state space model as described in Eq (5). This sensitivity function *S* represents the extent how the transfer function will be affected when one specific parameter changes. Because it is an MISO system, the sensitivity function *S* is a vector here. Then a Bode plot is used to display the sensitivity of every parameter.

### Simulation Procedure

Firstly, the complete numerical cardiovascular model is configured in Matlab/Simulink with normal physiological values, such as normal ventricular elastance value (representing ventricular contractility) and 60 bpm heart rate. Simulations are run on a computer with intel CORE i7 inside. Normal hemodynamics are obtained, of which the aortic flow, aortic pressure and venous pressure are used for the parameter identification process. It is noted that in the complete cardiovascular model, the model of the arterial system is the lumped five-element Windkessel model, of which the parameters are the normal values listed in Table 1. If the identification algorithm is good enough, it would yield nearly the same normal values listed in Table 1.

Next, the configuration of the complete numerical cardiovascular model is modified to simulate a parameter variation condition by setting the resistance and compliance of the systemic arterial system as ramp signals, respectively. Specifically, the value of resistance *R*_*sa*_ in the lumped five-element model is changed linearly from 1.0 *mmHg*∙*s*/*mL* to 1.3 *mmHg*∙*s*/*mL* during 50 seconds and then returned back to 1.0 *mmHg*∙*s*/*mL* after another 50 seconds maintenance. The compliance *C*_*sa*,1_ is changed in a similar way, with its value between 0.9 *mL*/*mmHg* and 0.8 *mL*/*mmH*. While all other parameters remain the same. The on-line identification algorithm should detect the parameter changes if it is good enough.

Additionally, white gaussian noise is added into the simulation data to simulate real clinical data with measurement noise. The resultant data is then used to validate the performance of the identification algorithm when noise exists. In this study, an additive white gaussian noise with a covariance value of 10 is chosen as the baseline intensity for all the relevant simulation data. Both simulations under the baseline intensity and triple baseline intensity are conducted and compared.

## Results

### Simulated hemodynamics

**Fig 4:**
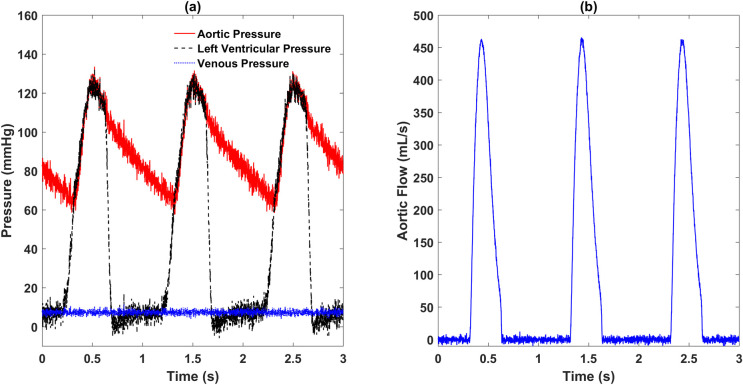
Simulated pressure and flow waveforms from the complete numerical cardiovascular model under the normal condition: (a) left ventricular pressure, aortic pressure and venous pressure, (b) aortic flow rate.

Basic hemodynamics of the cardiovascular model under the normal condition were obtained and are shown in Fig 4. The left ventricular pressure, aortic pressure, and venous pressure are depicted in Fig 4(A), while the aortic flow is shown in Fig 4(B). Normal left ventricular pressure ranged from near 0 *mmHg* to 130 *mmHg*, while the aortic pressure had a range of approximately 65–125 *mmHg*. The left ventricular pressure waveform was followed by the aortic pressure waveform well. The corresponding aortic flowrate shown in Fig 4(B), with a mean value of 84 *mL/s*, reached a peak value of 450 *mL/s* during heart ejection period and dropped to about 0 mL/s during diastole. The simulated hemodynamics is consistent with previous physiological reports.

**Fig 5:**
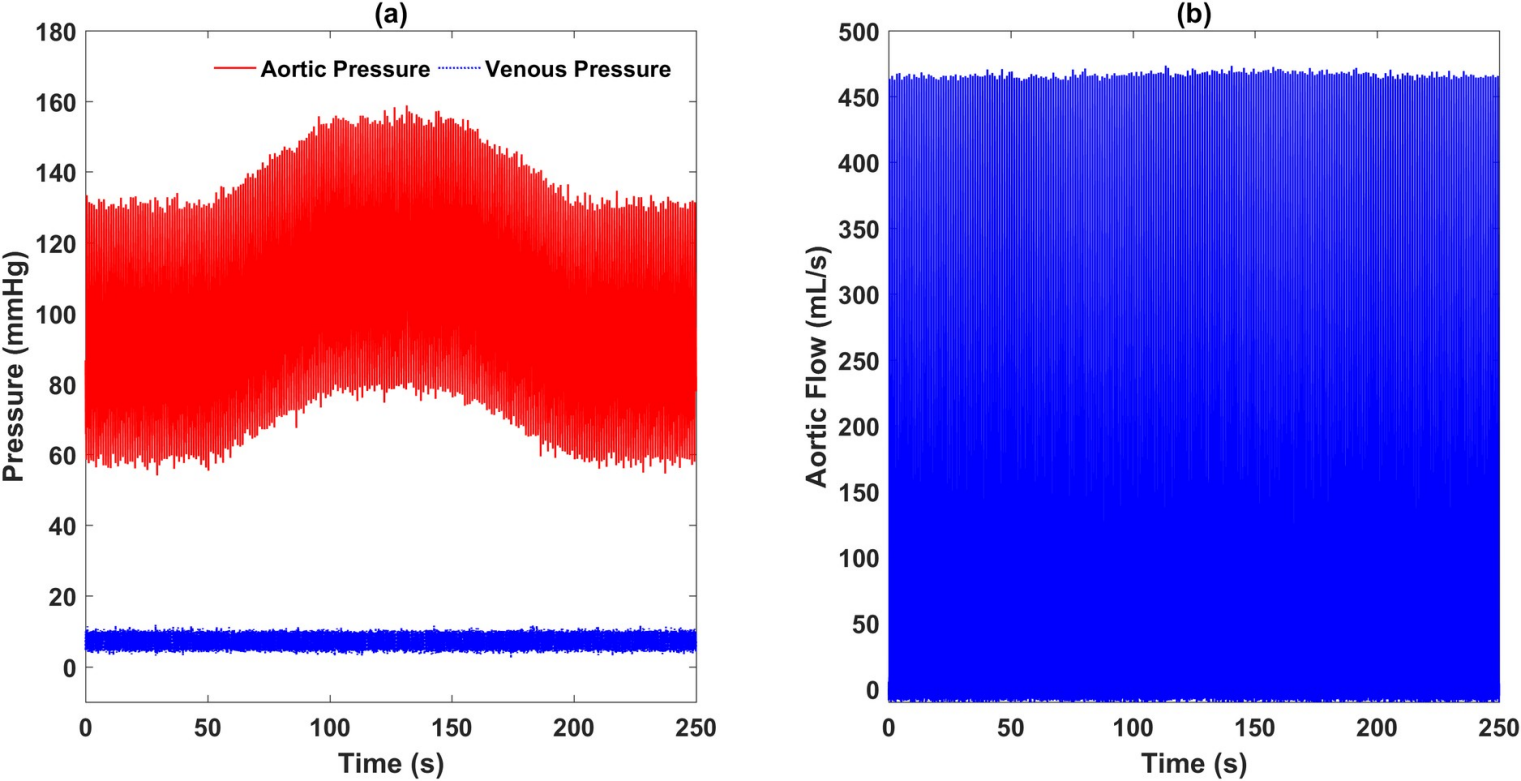
Simulated Pressure and flow waveforms during the variation of parameter values: (a) aortic pressure and venous pressure, (b) aortic flow rate.

Fig 5 shows the hemodynamics during the whole simulation when the resistance and compliance parameters of the systemic arterial system varies. The venous pressure and the aortic flow remained nearly unchanged. While the aortic pressure first increased to a range of about 80–155 *mmHg* during the period of 50–100 s, remaining unchanged for the next 50 seconds, and then decreased back to the previous level. This pressure variation is due to the value changes of the resistance *R*_*sa*_ and the compliance *C*_*sa*,1_.

### Parameter identification

**Fig 6:**
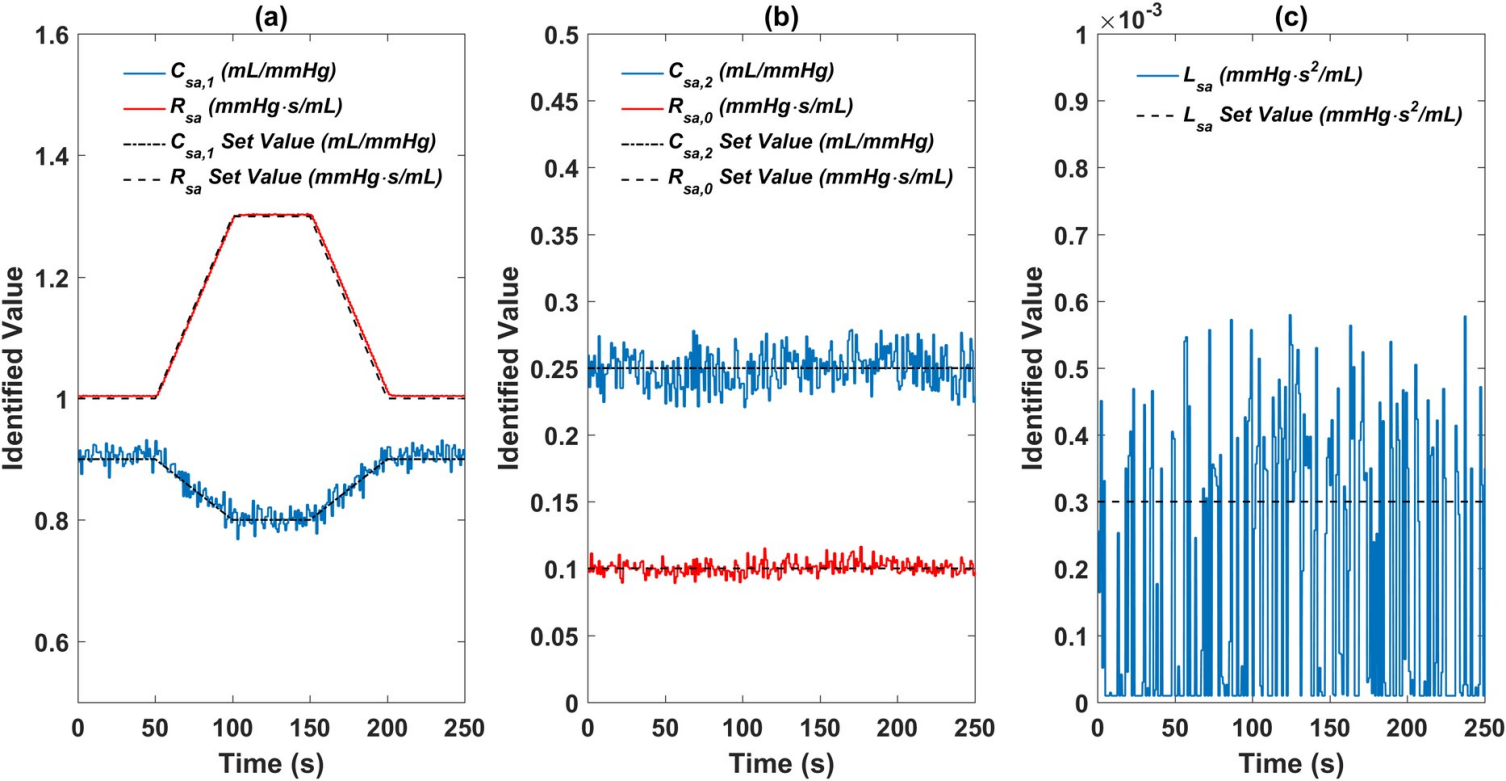
Plots of the on-line identified results of the five parameters. (a) ***R***_***sa***_ and ***C***_***sa*,1**_, (b) ***R***_***sa*,0**_ and ***C***_***sa*,2**_, (c) ***L***_***sa***_.

The on-line parameter identification process was performed all through the whole simulation. The identified values of the five parameters along with time are plotted in Fig 6. As shown in Fig 6(A), the identified values of the resistance *R*_*sa*_ and the compliance *C*_*sa*,1_ follow their set values very well. Compared to the compliance *C*_*sa*,1_, the identified result of the resistance *R*_*sa*_ exhibits a better performance. There is only a very small deviation between the identified values and the set ones of the resistance *R*_*sa*_ during the parameter variation, while the identified values of the compliance *C*_*sa*,1_ fluctuate obviously around the set values. To another resistance *R*_*sa*,0_ and compliance *C*_*sa*,2_, the identification results behave in a similar fluctuation way, as shown in Fig 6(B), however the fluctuation range of the resistance identified values is much smaller. The on-line identified values of the inertance *L*_*sa*_ have a range of 0.0001–0.0006 *mmHg*∙*s*^2^/*mL*, which show a large deviation from the set value 0.0003 *mmHg*∙*s*^2^/*mL*.

The mean and standard deviation for the identified values of the five parameters in the normal condition without parameter variation are listed in Table 2. It can be seen that the mean identified values fit the corresponding set values very well under both baseline intensity and triple baseline intensity noise conditions, which demonstrates the capability of the identification method to get the specific parameter values. However, there can be seen a slow trend of deterioration of the identification result when the noise intensity increases. Among all the parameters, the relative standard deviation for the parameter *R*_*sa*_ has the smallest value.

**Table 2:** Mean and standard deviation for the values of the on-line identified five parameters in the normal condition simulation.

Parameter	Original value [12]	Identified value statistical result (baseline intensity noise)		Identified value statistical result (3x baseline intensity noise)	
		Mean	Standard deviation	Mean	Standard deviation
*R*_*sa*,0_	0.1	0.100	0.005	0.100	0.007
*R*_*sa*_	1.0	1.004	0.0004	1.000	0.0000
*C*_*sa*,1_	0.9	0.906	0.013	0.907	0.019
*C*_*sa*,2_	0.25	0.249	0.014	0.249	0.014
*L*_*sa*_	0.0003	0.0002	0.0002	0.0001	0.0001

**Fig 7:**
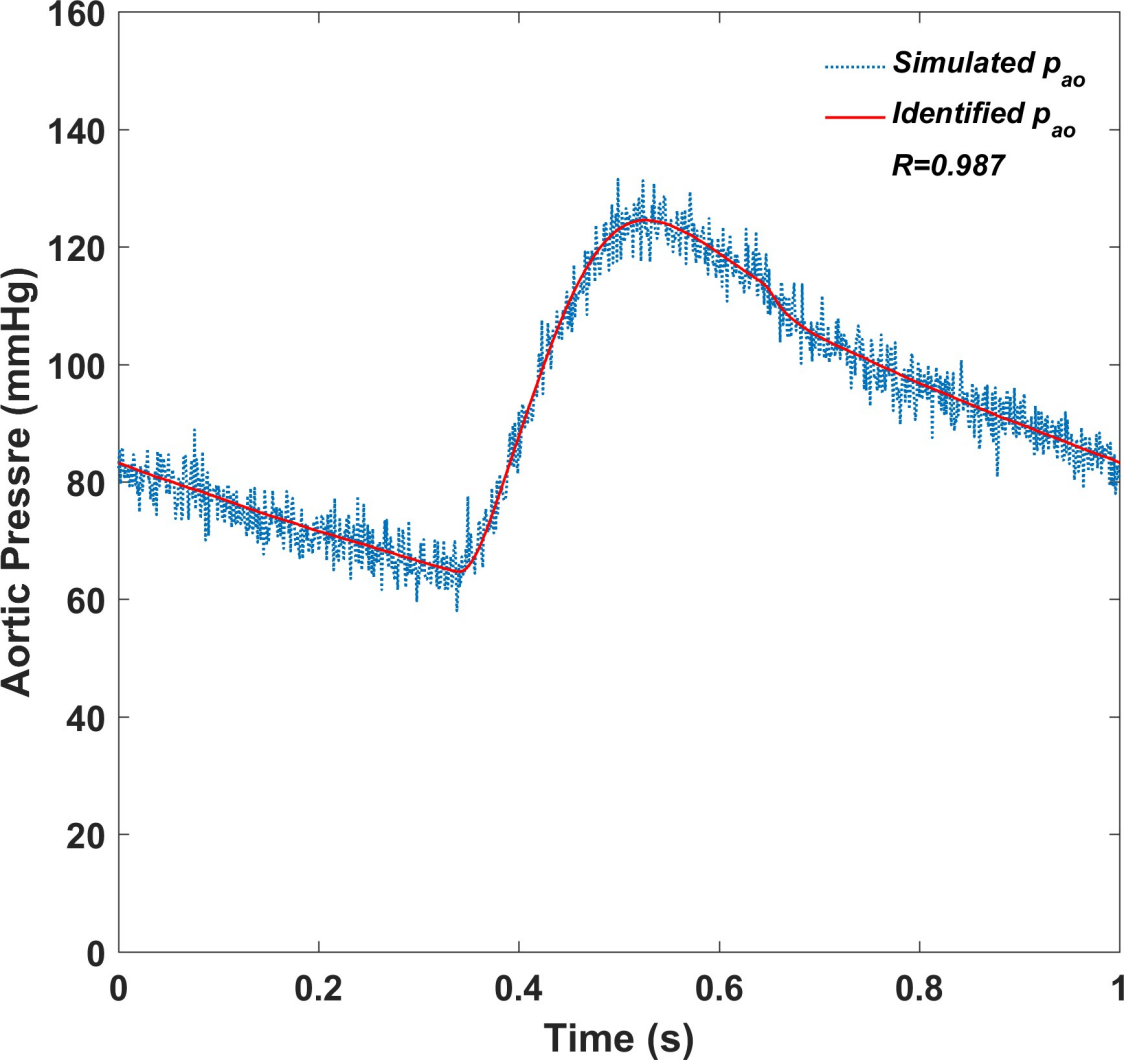
The predicted output of the lumped arterial model with identified parameter values and the simulated data from the numerical cardiovascular model.

Fig 7 shows how the predicted output of the five-element arterial model with identified parameter values fits the simulated data from the numerical cardiovascular model under the normal condition. Except for the additive white noise, the predicted output follows the simulated data very well, which demonstrates the accuracy of the identified values of the model parameters.

**Fig 8:**
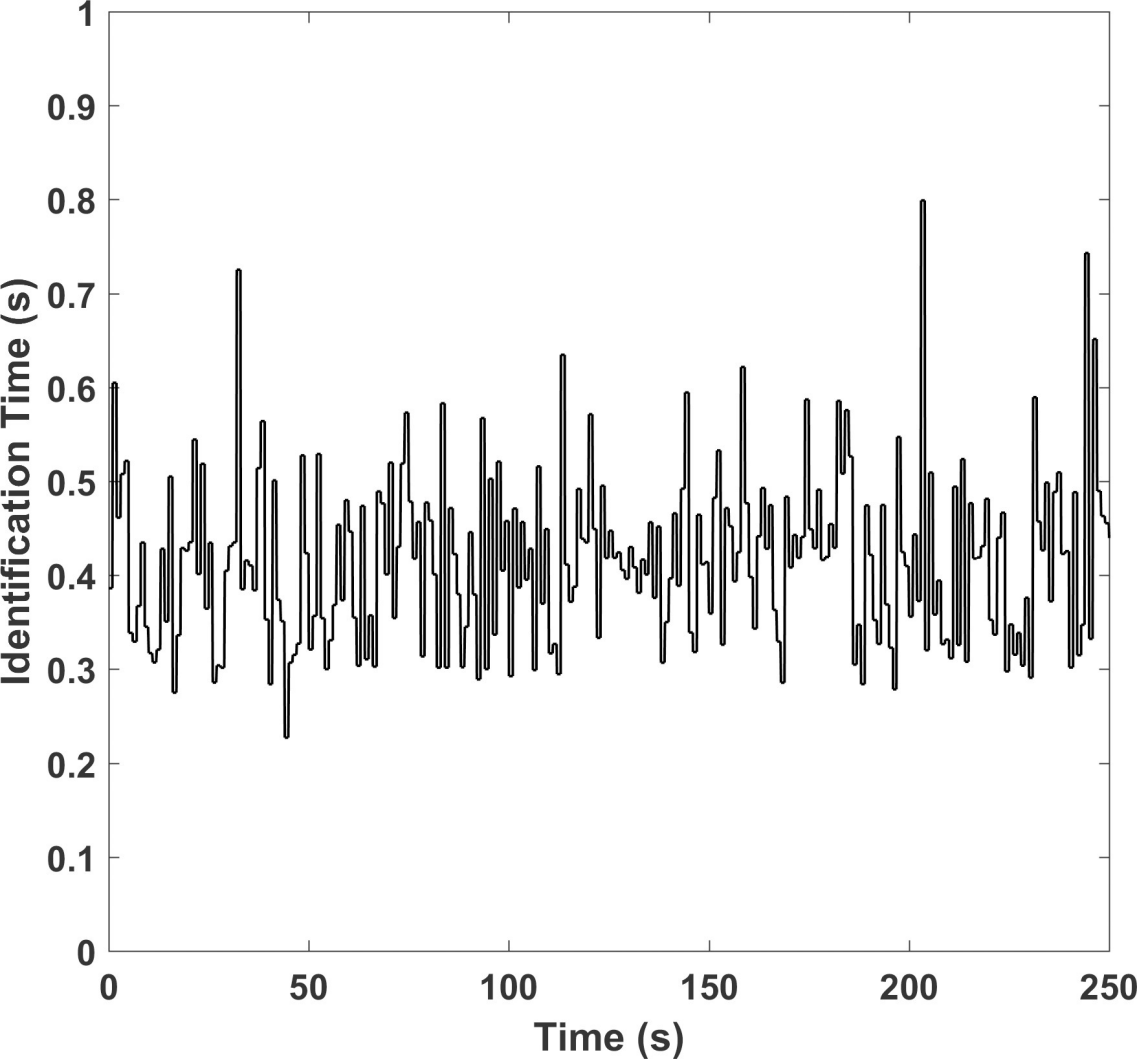
The parameter identification time for every cardiac cycle during simulation.

The parameter identification time for every cardiac cycle during simulation is plotted in Fig 8. It is clear that every identification time is less than 0.8 s, which is thought to be fast enough for the on-line identification application. It is noted that the genetic algorithm used to provide the first initial values costs about several minutes, however it is only carried out once at the beginning and will not affect the on-line identification afterwards.

### Parameter sensitivity

**Fig 9:**
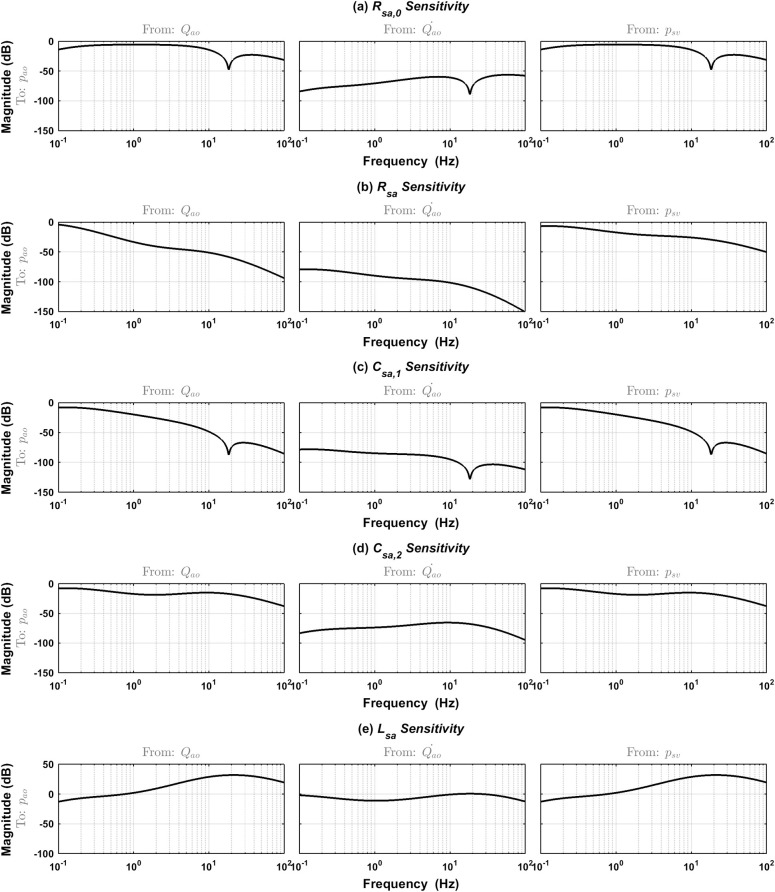
Bode plot of the parameter sensitivities of the model near the normal values in Table 1. Sensitivity to the parameters (a) ***R***_***sa*,0**_; (b) ***R***_***sa***_; (c) ***C***_***sa*,1**_; (d) ***C***_***sa*,2**_; (e) ***L***_***sa***_.

Parameter sensitivity analysis was carried out to see how changes of the model parameters affect output. In other words, understanding to which parameter the model is the most sensitive will help when analyzing the identification results. Fig 9 shows the Bode plot of the sensitivity function defined in Eq (12) for every model parameter. The fixed point where the sensitivity analysis was performed is Θ_0_ = [0.1 1.0 0.9 0.25 0.0003]^*T*^. The Bode plots are for every parameter from the multiple inputs to the output. The figure shows that the sensitivity from the inputs aortic flow *Q*_*ao*_ and venous pressure *p*_*sv*_ are the same for every parameter except the *R*_*sa*_ parameter. This is because for all the parameters except *R*_*sa*_, the difference of the sensitivity functions from the inputs *Q*_*ao*_ and *p*_*sv*_ is just the *R*_*sa*_ value, which equals to 1.0 in our analysis. At frequencies near the basic value of normal blood pressure waveforms in an adult, typically 1 Hz, the sensitivities to the parameters *R*_*sa*_,*C*_*sa*,1_,*C*_*sa*,2_ are small as their sensitivity functions *S* approach large negative values. The model is much more sensitive to *L*_*sa*_ for all inputs, and to *R*_*sa*,0_ for the first and third inputs. All the sensitivities at high frequencies decrease, except the parameter *L*_*sa*_, whose sensitivity increases. It is clear that the model sensitivity to the parameter *L*_*sa*_ is the most influential.

## Discussion

The good performance of the identification method proposed in this study demonstrates its effectiveness in determining the parameters of the lumped arterial system model online according to the related blood pressure and flow data. Although the validation for the identification method is implemented numerically, the cardiovascular model used is a good representative of the real blood circulation system, and the good performance of the method makes it reasonable to expect that this method may be applied to patient-specific data. This method has the potential use to monitor the arterial parameter variation of a specific patient.

Compared with a more complex arterial model, which usually models every main blood vessel branch with resistance, compliance and inertance, and then concatenated together to form a distributed arterial network model, the lumped arterial system model used in this research regards the arterial system as a whole. Due to the large parameter number of the distributed arterial network model, it will be difficult to make an accurate parameter identification, and hemodynamic measurements at more site of the arterial system will be demanded. More importantly, the time needed for the parameter identification of the distributed arterial network model would be a lot, making it impossible to be real time, while the identification of the lumped parameter in this research could be on line. As a result, if the distributed characteristics are not concerned and the rapid speed of parameter identification is required, the lumped arterial system model would be a more appropriate option.

The parameter identification of the five-element arterial system model proposed here is mainly aimed at customizing a HML served as a test platform for left ventricular assist devices. Our approach has advantages of simplicity and computational time savings. The identification process at each time point takes less than a second. It means that adjustments of parameters when human physiological state changes in a shorter period can be captured by this identification method; as a result, the HML could reproduce more physiological conditions by using time-dependent arterial model parameters. However, when considering other applications, such as testing an assist device with cannulation to a subclavian artery, it is necessary to adopt a more complicated arterial model with more branches and parameters. In short, the identification method with the five-element arterial model proposed here is suitable for applications where the arterial system could be regarded as a whole load.

It is noted that the on-line identification method is validated in the numerical cardiovascular model in this research. To apply this identification method in a specific patient in actual, measurements of the aortic pressure and flow rate, and the venous pressure should be carried out first. After that, the measured data will be fed into the on-line identification algorithm to find the specific parameter values in real time for this patient. These specific parameter values can then be used to customize a HML and find the best clinical solution such as the optimal speed of a ventricular assisted blood pump for this specific patient who will receive it. In this way, the HML is characterized for the specific patient by identification.

As just mentioned, blood pressure and flow rate measurements are necessary in actual use. The aortic and venous pressures could be obtained continuously and accurately by direct measurement using an intra-arterial catheter [16]. However, the catheterization is invasive and must be operated by a medical professional. Some indirect measurements such as the pressure pulse wave method have already been proposed [16,17], but the accuracy and dynamic performance should be improved further to be used for identification. For aortic flow measurement, the current clinical approach involves Doppler echocardiography, which monitors blood flow noninvasively in real time [18–20]. Additionally, an emerging technique using MRI also has potential usage for measuring the aortic flow [21]. Another concerning related with the clinical measurement is the measurement noise. It may deteriorate the performance of the parameter identification. On this account, the white noise was added in the numerical simulation, and the good results demonstrate the identification algorithm behaves still quite well. However, as implied by the statistical results in Table 2 under different intensity noises, a filtering preprocessing of the measured clinical data is still recommended before the parameter identification, especially for the data with low signal-to-noise ratio.

As shown in Fig 6 and Table 2, the identified results under baseline intensity noise of both the resistances *R*_*sa*_ and *R*_*sa*,0_ are obviously better than those of the other parameters. There exists a noteworthy deviation around the accurate value for either *C*_*sa*,1_ or *C*_*sa*,2_. The parameter *C*_*sa*,1_ has a maximum deviation of about 3.3%, while the parameter *C*_*sa*,2_ has a 10.4% maximum deviation. The maximum deviation of the parameter *L*_*sa*_ is nearly as large as its accurate value, but in terms of the absolute value this maximum deviation is still a very small value of only 0.0003 *mmHg*∙*s*^2^/*mL*. It can be concluded that it is more difficult to improve identification accuracy for parameters with smaller values.

According to the parameter sensitivity analysis, as shown in Fig 9, different frequency may affect the result. It may reveal a deteriorating trend of the result along with higher heart rate. Therefore, it is desirable to conduct more simulations under pathological conditions such as heart failure to validate the identification method further.

Although the study showed the validity of the on-line identification method, an improvement would involve further validation by applying the method to clinical data from a specific patient. Synchronous measurements of blood flow and pressures need to be performed to acquire the clinical data. In this case, the fit of the identified model would probably not be as accurate compared with the current study because of more complicated pressure, flow waveforms, and measurement noise. However, the identification has been good enough for actual use.

Apart from customizing a HML, another potential application of the on-line parameter identification method of the lumped arterial system model proposed in this research is the real time clinical monitoring of arterial vessel characteristics. It is important to monitor the characteristics such as resistance or compliance of the arterial vessel under some clinical circumstances, for example when a serious patient is after heart surgery or given the vascular-affecting drugs. Being aware of the arterial parameter value timely will be a good supplement to hemodynamic monitoring, which gives a direct and intuitive knowledge of the arterial blood vessel status of patients.

## Conclusions

In this study, an on-line parameter identification method for the five-element lumped arterial system model is proposed on the basis of iterative optimization. The method is implemented numerically on a complete cardiovascular model, and good results are yielded, proving its effectiveness in terms of good prediction and rapid convergence. Results also show that the on-line identification method can track the dynamic variation of the parameters very well. Furthermore, the parameter sensitivity analysis of the arterial model is included that elucidates the parameter effects on the model output. This method could be used to characterize a HML for a specific patient and other related systems such as real time clinical monitoring of arterial vessel characteristics.

## Supporting information

S1 Table Parameters of the cardiovascular model. List of all parameter values of the cardiovascular model used in simulation.
(DOCX)
