# Supplementary material for: On-line parameter identification of the lumped arterial system model: A simulation study
Source: PLoS One. 2020 Jul 10;15(7):e0236012. doi: 10.1371/journal.pone.0236012 (PMC7351215; doi:10.1371/journal.pone.0236012)
Supplement: S1 Table — List of all parameter values of the cardiovascular model used in simulation. (DOCX) [file pone.0236012.s001.docx]

# Supporting information

All parameter values of the cardiovascular model are listed in S1 Table 1.

**S1 Table 1. Parameters of the cardiovascular model.**

| **Symbol** | **Parameter** | **Value** | **Unit** |
| --- | --- | --- | --- |
| *C*_pa,1_ | Pulmonary compliance | 2 | mL/mmHg |
| *C*_pa,2_ | Pulmonary arterial compliance | 0.35 | mL/mmHg |
| *C*_sa,1_ | Aortic compliance | 0.9 | mL/mmHg |
| *C*_sa,2_ | Systemic arterial compliance | 0.25 | mL/mmHg |
| *C*_sv_ | Systemic venous compliance | 85 | mL/mmHg |
| *C*_pv_ | Pulmonary venous compliance | 10 | mL/mmHg |
| *L*_pa_ | Pulmonary arterial inertance | 0.002 7 | mmHg∙s^2^/mL |
| *L*_sa_ | Systemic arterial inertance | 0.000 3 | mmHg∙s^2^/mL |
| *L_p_*_v_ | Pulmonary valve inertance | 7.26×10^‒4^ | mmHg∙s^2^/mL |
| *L_t_*_v_ | Tricuspid valve inertance | 7.26×10^‒4^ | mmHg∙s^2^/mL |
| *L_a_*_v_ | Aortic valve inertance | 7.26×10^‒4^ | mmHg∙s^2^/mL |
| *L_m_*_v_ | Mitral valve inertance | 7.26×10^‒4^ | mmHg∙s^2^/mL |
| *R*_pa_ | Characteristic pulmonary resistance | 0.03 | mmHg∙s/mL |
| *R*_pvr_ | Pulmonary arterial resistance | 0.1 | mmHg∙s/mL |
| *R*_pv_ | Pulmonary venous resistance | 0.012 | mmHg∙s/mL |
| *R*_sa,0_ | Characteristic systemic resistance | 0.1 | mmHg∙s/mL |
| *R*_sa_ | Systemic arterial resistance | 1.0 | mmHg∙s/mL |
| *R*_sv_ | Systemic venous resistance | 0.081 | mmHg∙s/mL |
| *R*_rv_ | Right ventricle internal resistance | 0.03 | mmHg∙s/mL |
| *R*_lv_ | Left ventricle internal resistance | 0.1 | mmHg∙s/mL |
| *R*_pv_ | Pulmonary valve direct resistance | 0.003 75 | mmHg∙s/mL |
| *R*_tv_ | Tricuspid valve direct resistance | 0.003 75 | mmHg∙s/mL |
| *R*_av_ | Aortic valve direct resistance | 0.003 75 | mmHg∙s/mL |
| *R*_mv_ | Mitral valve direct resistance | 0.007 5 | mmHg∙s/mL |

# References

1. Colacino FM, Moscato F, Piedimonte F, Arabia M, Danieli GA. Left Ventricle Load Impedance Control by Apical VAD Can Help Heart Recovery and Patient Perfusion: A Numerical Study. ASAIO J. 2007;53: 263–277. doi:10.1097/MAT.0b013e31805b7e39.
2. Huang F, Gou Z, Fu Y, Ruan X. Effects on the pulmonary hemodynamics and gas exchange with a speed modulated right ventricular assist rotary blood pump: a numerical study. Biomed Eng Online. 2018;17: 142. doi:10.1186/s12938-018-0591-4.
